# Assessment the effect of diabetes education on self-care behaviors and glycemic control in the Turkey Nursing Diabetes Education Evaluating Project (TURNUDEP): a multi-center study

**DOI:** 10.1186/s12912-022-01001-1

**Published:** 2022-08-05

**Authors:** Selda Celik, Nermin Olgun, Feride Taskin Yilmaz, Gulden Anataca, Ilksen Ozsoy, Nurcan Ciftci, Elif Fidan Aykiz, Serap Yasa, Ebru Karakiraz, Yeliz Ulker, Yeliz Erdem Demirhan, Sultan Yurtsever Celik, Inci Arpaci, Fulya Gunduz, Derya Temel, Cevahir Dincturk, Betul Essiz Sefer, Elif Bagdemir, Esin Erdem, Esra Sarimehmetoglu, Fatime Sahin, Gulay Gulsen, Nese Kocakgol, Sibel Gokmen, Suna Damar, Zekiye Celikoz, Yesim Korkusuz, Senay Kirlak, Tugce Dede, Behice Kahraman, Arzu Sert, Nesrin Cetin

**Affiliations:** 1 Hamidiye Faculty of Nursing, University of Health Sciences Turkey, Istanbul, Turkey; 2 Mekteb-I Tıbbiye-I Şahane (Hamidiye), Külliyesi Selimiye Mahallesi Tıbbiye Caddesi No:38 34668 Üsküdar, Istanbul, Türkiye; 3 Faculty of Health Sciences, Hasan Kalyoncu University, Gaziantep, Turkey; 4 Faculty of Health Sciences, Sakarya University of Applied Sciences, Sakarya, Turkey; 5 University of Health Sciences Turkey, Kanuni Sultan Suleyman Training and Research Hospital, Istanbul, Turkey; 6 Istanbul Haydarpasa Numune Training and Research Hospital, Istanbul, Turkey; 7 Kastamonu State Hospital, Kastamonu, Turkey; 8 Kocaeli SEKA State Hospital, Kocaeli, Turkey; 9 Canakkale Mehmet Akif Ersoy State Hospital, Canakkale, Turkey; 10 Sinop Boyabat 75, Yil State Hospital, Sinop, Turkey; 11 25 Aralık State Hospital, Gaziantep, Turkey; 12 Faculty of Medicine Hospital, Kocaeli University, Kocaeli, Turkey; 13 University of Health Sciences Turkey, Bagcılar Training and Research Hospital, Istanbul, Turkey; 14 Şehit Kamil State Hospital, Gaziantep, Turkey; 15 Antalya Kepez State Hospital, Antalya, Turkey; 16 Eskisehir Yunus Emre State Hospital, Eskisehir, Turkey; 17 Eskisehir State Hospital, Eskisehir, Turkey; 18 Kartal Kosuyolu Yuksek Ihtisas Training and Research Hospital, Istanbul, Turkey; 19 Istanbul Faculty of Medicine, Istanbul University, Istanbul, Turkey; 20 Usak Training and Research Hospital, Usak, Turkey; 21 Bartin State Hospital, Bartin, Turkey; 22 Karabuk Training and Research Hospital, Karabuk, Turkey; 23 Ondokuz Mayıs University Hospital, Samsun, Turkey; 24 Gaziantep Dr. Ersin Arslan Training and ResearchHospital, Gaziantep, Turkey; 25 Urla State Hospital, Izmir, Turkey; 26 Umraniye Training and Research Hospital, Istanbul, Turkey; 27 Kutahya Evliya Celebi Training and Research Hospital, Kutahya, Turkey; 28 Hamidiye Faculty of Medicine, University of Health Sciences Turkey, Istanbul Kartal Dr. Lutfi Kirdar City Hospital, Istanbul, Turkey; 29 Acibadem Maslak Hospital, Istanbul, Turkey; 30 Turkish Diabetes Association, Istanbul, Turkey; 31 Isparta City Hospital, Isparta, Turkey

**Keywords:** Type 2 diabetes, Diabetes nurse, Patient education, Self-care, Glycemic control

## Abstract

**Background:**

Diabetes education in Turkey is provided by diabetes nurse educators in almost all healthcare organizations. However, the education is not standardized in terms of learning content, duration, and methods. This multi-center study was performed to assess the self-care behaviors and glycemic control following education provided to the patients with type 2 diabetes mellitus by diabetes nurse educators.

**Methods:**

This was a descriptive and cross-sectional study and included 1535 patients admitted to 28 public hospitals for the treatment of type 2 diabetes mellitus. The education was assessed by using a Patient Identification Form and Self-care Scale.

**Results:**

The proportion of individuals who received diabetes education within the last year was 78.5%, with 46.7% of them having received it once. Of the patients, 84.8% reported that they received diabetes education individually. It was found that the proportion of individuals who received education about oral antidiabetics (78.5%) and glucose testing at home (78.5%) was higher than the proportion of individuals who received education about exercise (58.8%) and foot care (61.6%). The status of diabetes education, education intervals, and the correlation of the education method with self-care and glycemic control was evaluated. Self-care and glycemic control levels were better among the patients who received diabetes education thrice or more and in patients who received education both individually and in a group (p < 0.05).

**Conclusions:**

Approximately three-quarters of individuals with type 2 diabetes mellitus received education by diabetes nurse educators in Turkey. Diabetes education is positively correlated with self-care and glycemic control levels among patients with type 2 diabetes mellitus. Efforts for generalization and standardized education for all diabetes patients are necessary.

## Background

While diabetes mellitus is not contagious, it is a gradually increasing health problem due to its prevalence and related complications [1]. Diabetes is one of the most rapidly growing global health conditions of the twenty-first century due to the rate of population growth and increasing urbanization. Diabetes mellitus has affected more than half a billion people between the ages of 20 and 79 years globally. While the prevalence of diabetes mellitus in Turkey was 8.1% in 2011, it increased to 14.5% in 2021. It is predicted that Turkey will be among the top ten countries in the world in terms of diabetes prevalence in 2045 [2].

Since diabetes mellitus is a cause of concern due to its high prevalence and direct effects on mortality, disability, and healthcare expenses, it is critically important to control and effectively manage the disease [3]. In this context, while increasing awareness about the prevention of diabetes mellitus is the primary goal, making and maintaining lifestyle changes from the time of diagnosis is one of the important approaches [4]. For effective disease management in patients diagnosed with diabetes mellitus, it is important that patients are informed about the disease, learn about the appropriate use of medication, adapt their diet and physical activities according to the disease condition, and adopt behaviors such as self-monitoring and regular medical follow-up and care [4, 5]. It is important to improve the individual's self-care for the adoption and implementation of these behaviors [6].

The most effective method for improving self-care in individuals with diabetes mellitus is diabetes education [7]. Diabetes education is one of the effective interventions to influence lifestyle changes and is an integral part of the treatment for diabetes. Diabetes education aims to ensure the effective participation of individuals with diabetes mellitus by improving their compliance to treatment, preventing the complications associated with diabetes, reducing the cost of treatment, and improving the individual’s quality of life [5]. In this context, diabetes education includes sharing knowledge and experience to ensure that the individuals with diabetes feel better, can prevent possible complications by better control of the disease, reduce the cost of treatment, reduce treatment-related errors, and ensure that the patients adopt emerging technologies [7, 8]. Studies have demonstrated that diabetes education improves the self-care behavior of individuals, glycemic control, and quality of life [7, 9–12]. Similarly, diabetes education helps people adopt a positive attitude toward the disease and improves treatment compliance [13]. Moreover, diabetes education reduces hospital and emergency room admissions and healthcare expenses [14, 15].

Healthcare professionals, especially nurses, have an important responsibility to meet the educational requirements of individuals with diabetes mellitus [16]. The importance of the nurses’ role in educating individuals with diabetes mellitus is known globally [17]. Most of the education and support to individuals with diabetes mellitus, both in community and acute-care hospital settings in many regions of Europe, Australia, New Zealand, and the USA, is provided by specialist nurses [18]. In Turkey, diabetes education is predominantly provided by diabetes nurse educators.

In addition to providing education, diabetes nurse educators support patients to set their goals for diabetes control, develop problem-solving skills, improve their knowledge and skills about self-care activities, gain self-confidence and self-competence, manage their own responsibilities, and be involved in the decision-making process [1]. Moreover, nurses play an important role in identifying the patients’ needs for education and improving the patients’ skills in controlling diabetes [8]. It has been reported that compared to other healthcare professionals, nurses have a higher possibility of encouraging healthcare-seeking behaviors [19]. Similarly, it has been highlighted that nurses can provide effective and qualified care at lower costs; therefore, nurses should play an important role in the education about diabetes treatment and care [17].

Diabetes education is provided by diabetes nurse educators to out-and in-patients with diabetes mellitus in almost all hospitals in Turkey, and this education is provided as a group activity within the scope of the ‘Patient/Diabetes School’ program rolled out by the Ministry of Health Hospitals. Additionally, diabetes nurse educators provide group education using slide shows prepared based on their own literature search or individual education using the demonstration and narration method. However, the evidence about the efficacy of these educations provided by diabetes nurse educators is inconclusive at present. There is no study demonstrating the efficacy of diabetes education routinely provided by diabetes nurse educators in Turkey. This study is important because it reveals the effect of the education provided by diabetes nurse educators using different methods and for various durations on the self-care behaviors and glycemic control of the patients. Furthermore, it is anticipated that this study will contribute to the literature in terms of demonstrating the efficacy of diabetes education for the benefit of the patients.

## Methods

### The aim and design of the study

TURNUDEP study (TURkey NUrsing Diabetes Education Evaluating Project) is designed as a multi-center, descriptive, and cross-sectional study aiming to determine the effect of diabetes education on self-care behaviors and glycemic control provided to individuals with type 2 diabetes mellitus (T2DM) by diabetes nurse educators in Turkey.

### Study setting and sample

Before starting the research, an announcement was made and all diabetes nurses who were members of the Diabetes Nursing Association were informed about the research. Subsequently, the research was conducted in 28 hospitals with nurses who volunteered to participate in the research and had diabetes nurse educator certificates provided by the Ministry of Health. Individuals with T2DM who visited the internal medicine, diabetes, and endocrinology outpatient clinics of these hospitals between April 15 and October 15, 2021, constituted the study population. The sample size for the study was calculated using power analysis. According to the self-care variable in the reference study [7], the total number of patients required to achieve a significance level of 0.05, an effect size of 0.4, an error risk of 0.05, and a power of 1-β = 0.95 was determined as 71. Due to the multi-centric study design, it was envisaged that at least 25 patients per hospital would be included in the study. Finally, 1535 patients who had T2DM for at least 6 months, had no verbal communication barriers, completed the data forms, and agreed to participate in the study were included. However, 43 individuals with T2DM whose data was missing in the forms were excluded from the study.

### Data collection tools

Data were collected using a patient identification form and Self-care Scale.

The patient identification form was prepared by the research team and consisted of two sections. The first section included the socio-demographic, disease and glycemic control parameters (fasting blood glucose and HbA1c), and the second section included the diabetes education information.

#### Diabetes self-care scale (DSCS)

The scale was developed by Lee and Fisher to evaluate the self-care activities of patients with T2DM [20]. It is a 4-point Likert-type scale and consists of 35 items. The scale aims to collect information on eating time and status, exercise status, blood glucose monitoring and recording status, use of oral antidiabetics and insulin as recommended, doctor visits for blood glucose control, foot care, personal hygiene practices, and diabetes and its complications. Patients with a score above 66% (92.4 points) are considered to have an acceptable level of self-care. The scale scores range between 35 and 140, with higher scores having a positive correlation with patients’ self-care abilities. The validity and reliability study of the scale for Turkey was performed by Karakurt and Kaşıkçı, and Cronbach’s α reliability coefficient was found to be 0.81 [7]. In this study, Cronbach’s alpha coefficient of the scale was found to be 0.91.

### Routine practice

Diabetes education is predominantly provided by diabetes nurse educators in Turkey. However, it is not standardized in terms of learning content, duration, and methods. While diabetes nurses use the education set provided by the Ministry of Health, they also prepare content based on the literature. In general, the learning content includes the description, treatment, complications, and home monitoring of the disease. Patients receive individual or group education through face-to-face standard narration and demonstration methods. At the end of each session, an appointment is made for the next session, and multiple sessions might be provided to each patient who returns for the appointment or requests.

Data were collected by the investigators on the day when the patients visited the outpatient clinic for their appointment through an interview in an empty clinic room. The height, weight, and blood pressure of the patients were measured by the investigators using measuring instruments in the study centers.

Before initiation of the study, diabetes nurse educators who participated as researchers were instructed about the methods to take the measurements to ensure standardization in height, weight, and blood pressure measurements. Data about the chronic complications were obtained from the patient records. Measurements and completion of the data forms by the investigators required approximately 25 to 30 min.

### Data evaluation

SPSS 22.0 software was used to analyze the study data. The normality of distribution of the data was tested using the Kolmogorov–Smirnov test. Percentages and means were used for analyzing the distribution of the socio-demographic, disease, treatment, glycemic control parameters, and diabetes education characteristics of the participants. For the comparison of the diabetes education characteristics and mean DSCS scores of the patients, the student’s t-test and Mann–Whitney U test were used for the homogeneous data and the Kruskal–Wallis test for the non-homogeneous data. Post-hoc analysis was used to compare multi-groups that the difference between them was statistically significant. Furthermore, Pearson’s correlation analysis was used to compare the mean DSCS scores and mean HbA1c values. A statistical significance level of 0.05 was considered.

## Results

The mean age of the patients was 56.19 ± 11.63 years, with 73.3% of them aged < 65 years. Of the total participants, 54.7% were females, 86.9% were married, and 45.5% were primary school graduates. Sixty-three point eight percent of the patients were unemployed, and almost all of them (97.3%) had social security, with 61.5% of them reporting to be from middle-income families. Seven point three percent were living alone, and 82.1% of them were living with their spouse and/or children; 25.4% reported their general health status as good, while 55.8% reported it was moderate. Of the total patients, 20.5% were current smokers; their mean cigarette count per day was 19.16 ± 12.48, and their average smoking duration was 19.69 ± 11.68 years. Seven point six percent reported consuming alcohol; 6.2% consumed it very rarely, 1.3% consumed frequently, and 0.2% consumed it daily. On analysis of the body-mass index (BMI), 36.9% of the patients were found to be overweight (BMI = 25–30 kg/m^2^), and 45.3% of them were obese (BMI > 30 kg/m^2^). The mean duration of diabetes in the patients was 10.25 ± 7.48 years. Table 1 shows the disease characteristics of the participants.

**Table 1:** Disease characteristics of individuals with Type 2 diabetes mellitus

Characteristics	n	%
**Disease duration (years) (Mean ± SD)**	10.25 ± 7.48	
Fasting blood glucose (mg/dL)	208.77 ± 92.30	
HbA1c (%)	9.56 ± 2.24	
**Treatment method**		
Oral antidiabetic	415	27.0
Oral antidiabetic plus insulin	887	57.8
Insulin	233	15.2
**Duration of insulin treatment (years) (Mean ± SD)**	5.32 ± 4.32	
**The number of patients with emergency room visits due to diabetes within the last year**	356	23.2
**The frequency of emergency room visits due to diabetes within the last year**^**a**^		
Once	244	68.5
Twice	62	17.4
Three times or more	50	14.1
**The reason for emergency room visit**^**a**^		
Hyperglycemia	266	74.5
Hypoglycemia	31	8.7
Chronic diabetes complication	36	10.4
Infection	23	6.4
**The number of patients hospitalized due to diabetes within the last year**	238	15.5
**The reason for hospitalization**^**a**^		
Hyperglycemia/Blood glucose regulation	192	80.6
Hypoglycemia	3	1.3
Diabetes complication	23	9.7
Infection	20	8.4
**The frequency of hospitalizations**^**a**^		
Once	176	74.0
2–3 times	40	16.8
4 times or more	22	9.2
**The number of hospitalization days (Mean ± SD)**	9.38 ± 7.43	
**The number of patients with a family member/relative with diabetes**	1067	69.5
**The degree of the relative with diabetes**^**a**^		
First degree relatives	567	53.1
Second degree relatives	369	34.6
Spouse	131	12.3
**The number of patients who return for diabetes follow-up**	852	55.5
**The frequency of diabetes follow-up visits**^**a**^		
Monthly	312	36.7
Quarterly	324	38.1
Annually	85	13.0
Irregular	77	9.1
When they feel unwell	52	6.1
**The number of patients with a chronic disease other than diabetes**	959	62.5
** Presence of neuropathy**	462	30.1
** Presence of retinopathy**	289	18.8
** Presence of nephropathy**	126	8.2
** Presence of diabetic foot**	90	5.4
** Presence of high blood pressure**	735	47.9
** History of myocardial infarction**	200	13.0
** Presence of cerebrovascular accident**	54	3.5

^a^ “n” count is changed. *SD* Standard deviation

The proportion of the patients who received education from the diabetes nurse educator within the last year was 78.5%. Forty-six point seven percent of the participants received diabetes education once, 27.7% received it twice, and 25.6% received it thrice or more. Eighty-four point eight percent of the subjects received individual education, 2.2% received group education, and 13.1% received both individual and group education. The most frequent education provided to the patients was about oral antidiabetic agents and home blood glucose monitoring (78.5%), while the least frequently provided education was about exercise (58.8%) and foot care (61.6%). It was observed that individual education is more common among all education subjects (Table 2).

**Table 2:** Diabetes education characteristics of individuals with Type 2 diabetes mellitus

Education subjects	Education status				Education method					
	**Received**		**Not received**		**Individual**		**Group**		**Both**	
	**n**	**%**	**n**	**%**	**n**	**%**	**n**	**%**	**n**	**%**
Diabetes in general	1205	78.5	330	21.5	1022	84.8	26	2.2	157	13.1
At-home blood glucose monitoring	1205	78.5	330	21.5	1119	92.9	8	0.6	78	6.5
Oral antidiabetic agent	1205	78.5	330	21.5	1118	92.8	9	0.7	78	6.5
Hypoglycemia	1178	76.7	357	23.3	1063	90.3	12	1.0	103	8.7
Diet	1172	76.4	363	23.6	1064	90.8	14	1.2	94	8.0
Insulin	1090	71.0	445	29.0	1014	93.0	6	0.6	70	6.4
Hyperglycemia	1070	69.7	465	30.3	957	89.5	10	0.9	103	9.6
Diabetic foot	946	61.6	589	38.4	851	90.0	13	1.3	82	8.7
Exercise	898	58.8	637	41.2	780	86.9	36	4.0	82	9.1

The mean DSCS score was 83.46 ± 16.22, with 28.3% of the patients having an acceptable level of self-care based on the cutoff value of the scale. Table 3 shows information about the diabetes education of the patients and a comparison of the self-care and HbA1c levels. It shows that education status, frequency, and method are correlated with self-care and glycemic control (p < 0.05). Accordingly, it was observed that the patients who received diabetes education had better self-care and glycemic control than those who did not. In the post-hoc analysis, it was found that the self-care levels of patients who received education more than twice and those who received both individual and group education were better. In addition, it was found that the glycemic control levels of patients who received education thrice or more and those who received both individual and group education were better.

**Table 3:** Diabetes education characteristics of the patients and the comparison of their self-care and HbA1c levels

	**Diabetes Self-Care Scale**	**HbA1c**
Diabetes education		
Received	86.51 ± 15.34	9.46 ± 2.23
Not received	72.34 ± 14.60	9.82 ± 2.27
t, p	15.044; p < 0.01	-2.329; 0.020
The frequency of diabetes education		
Once^a^	82.42 ± 15.60	9.80 ± 2.43
Twice^b^	88.89 ± 14.51	9.49 ± 2.03
Three times or more^c^	90.90 ± 14.39	9.00 ± 1.96
F, p	37.845; p < 0.01	12.795; p < 0.01
Difference	b > a, c > a	a > c
The method of diabetes education		
Individual^a^	85.29 ± 15.06	9.62 ± 2.26
Group^b^	86.71 ± 12.05	8.99 ± 2.20
Both^c^	94.29 ± 15.78	8.76 ± 1.92
KW, p	44.336; p < 0.01	22.260; p < 0.01
Difference	c > a, c > b	a > c

All results are expressed as mean ± SD, unless otherwise stated. SD, standard deviation

*t* Student’s t-test, p *p* value, *F* Mann–Whitney U test, *KW* Kruskal–Wallis test

## Discussion

Diabetes is a chronic disease requiring routine and complex individual care [21]. Diabetes education comprises sharing knowledge and skills for controlling the glycemic level to prevent long-term complications and is a valuable strategy to improve the health behaviors of individuals with diabetes mellitus [22, 23]. The present multi-centric study analyzed the effects of education provided to individuals with T2DM by diabetes nurse educators in Turkey and found that the proportion of patients who received education from a diabetes nurse educator within the last year was 78.5%. The study results showing that the diabetes nurse educators who aspire to reach all individuals with diabetes were able to provide education to three out of four patients admitted to healthcare facilities is an encouraging finding. However, the inability to reach all individuals might be related to the lack of diabetes nurse educators in the facilities and the referral being at the discretion of the physician.

In diabetes management, continuity and repetition of the education are as important as the education itself [14, 24]. The present study revealed that almost half of the patients (46.7%) received education only once. The follow-up and treatment of individuals with diabetes mellitus are usually done in outpatient clinics in hospital settings in Turkey. Usually, individuals with diabetes do not regularly return for follow-up visits; hence, they cannot be monitored by healthcare professionals [24]. Although patients are given appointments for further education after the initial education provided by the diabetes nurse educator, patients’ participation in repetitive education might be low. This might be due to the low education level of the patients. In this study, almost half of the patients (45.5%) were primary school graduates. The low level of education might negatively impact the belief about the importance of diabetes education. In addition, the cost of transportation to the hospital might be one of the reasons that reduce participation in repetitive education. In this study, the economic level of approximately half of the patients (55%) was moderate. In general, the findings of the study are important because they show that participation in repetitive education is low in Turkey.

The study revealed that a vast majority of the individuals with diabetes mellitus (84.8%) receive individual education, while the proportion of the individuals receiving group education is low (2.2%). Another study showed that 85.1% of the educational sessions provided by the nurses were individual sessions [13]. It has been reported that compared to individual education, group education has a better effect on improving diabetes health outcomes and is also more economical [25]. A study showed that group education is effective in improving glycemic control and knowledge about diabetes in individuals with T2DM [26]. In contrast, another study showed that individualized diabetes education is more effective than group education in facilitating the control of T2DM [9]. In Turkey, group education is provided at many healthcare facilities under the patient school program. The high proportion of the patients receiving individual education in this study might be because fewer group education sessions were planned due to the COVID-19 pandemic.

For successful disease control and treatment of an individual diagnosed with diabetes mellitus, it is important to provide education about the treatment options, the importance of diet and exercise, blood glucose level monitoring, managing unexpected situations, and recognizing and preventing complications [1]. Currently, in-hospital diabetes education models focus only on survival skills, including hypoglycemia, medication training, diet, and blood glucose monitoring [15]. This study demonstrated that patients are most frequently provided education about oral antidiabetic agents and blood glucose monitoring at home (78.5% for both), while education on exercise (58.8%) and foot care (61.6%) is least frequent. In another study, it was highlighted that the learning content provided by the nurses to individuals with diabetes mellitus is consistent with the literature; however, it is lacking in terms of acute and chronic complications of diabetes [13]. In a study investigating the diabetes education provided by in-clinic nurses in Turkey, it was found that the first three education topics covered by the nurses are “the importance of the insulin treatment, insulin injection regions, injection site rotation, side effects of the insulin treatment and insulin storage” (16.0%), “definition and use of the antidiabetic agents” (14.0%), and “the definition and symptoms of diabetes” (13.8%) [27]. Different findings of the studies might be due to the lack of standardized education content provided by the diabetes nurse educators.

Successful diabetes management requires lifelong adherence to self-care [28]. Self-care involves individuals taking necessary steps to sustain their lives, health, and well-being. To control their disease, individuals with diabetes mellitus should adopt self-care activities, including proper diet, regular exercise, blood glucose control, appropriate use of oral antidiabetics, recognizing the effects and adverse effects of insulin treatment, avoiding smoking and alcohol, preventing complications of diabetes, lifelong adherence to anti-diabetes treatment, and attending follow-up visits [1, 10, 16]. Diabetes education is essential for improving self-care in individuals with diabetes [7]. The present study revealed that patients receiving diabetes education have higher levels of self-care. Many studies have also shown that education has a positive effect on self-care in individuals with diabetes mellitus [7, 11, 19, 29]. However, a study reported that although individuals with diabetes who received diabetes education had high self-care level, there was no significant difference between them when compared to the group who did not receive education [30]. Among the in-patients, diabetes self-management education and support programs were conducted to improve the knowledge and treatment compliance of patients under the leadership of the nurses. An increase in the knowledge about diabetes among patients was seen; however, no change was found in their treatment compliance [31]. The present study shows that diabetes education provided by nurses is beneficial in improving the level of self-care.

The aim of diabetes education is to achieve glycemic control and prevent complications. Diabetes education creating awareness about the disease is essential to achieve effective glycemic control [32]. The present study demonstrated that patients receiving diabetes education have better glycemic control levels. The study results are consistent with those reported in the literature. Many studies have reported that individuals with diabetes receiving education have better glycemic control [7, 11, 12, 19, 22, 28, 32–35]. In the present study, it was observed that education provided by diabetes nurse educators contributes to disease management. The study finding is important as it emphasizes that education is an essential part of the disease management of individuals with diabetes.

This study has some limitations. While the study was performed in 28 public hospitals located in different regions of Turkey, the results cannot be generalized to the general population. In addition, individuals with T2DM were randomly included in the study by the diabetes nurse educators who conducted the study. Since it is a cross-sectional, questionnaire-based study, it has an inherent limitation of time. The data about the education of subjects and self-care levels of the individuals with T2DM are self-reported. Additionally, the inability to monitor diabetes education due to the lack of a patient follow-up system is an important limitation.

## Conclusions

The present study found that diabetes nurse educators provide diabetes education to approximately three-fourths of the patients in Turkey. Furthermore, it was found that diabetes education is positively correlated with self-care and glycemic control levels among individuals with T2DM. The present study once again emphasized the importance of the diabetes education provided by the nurses though it is not standardized. Nevertheless, practices such as establishing a follow-up system, taking steps to improve the efficiency of the appointment system, establishing and following a standardized diabetes education program under the guidance of the Ministry of Health in Turkey, and increasing the number of diabetes nurse educators in health facilities are necessary to improve the efficiency of the education. In addition, randomized controlled studies evaluating the effects of age, disease duration, and other characteristics on self-care and glycemic control in patients receiving diabetes education are necessary. Further studies are recommended to determine the factors that might affect the efficiency of education provided by diabetes nurse educators.

## Data Availability

The datasets used and/or analyzed during the current study are available from the corresponding author on reasonable request.

## Abbreviations

BMI: Body-Mass Index
COVID-19: Coronavirus Disease 2019
DSCS: Diabetes Self-Care Scale
T2DM: Type 2 Diabetes Mellitus
TURNUDEP: TURkey NUrsing Diabetes Education Evaluating Project
